# Visualizing insulin vesicle neighborhoods in β cells by cryo–electron tomography

**DOI:** 10.1126/sciadv.abc8258

**Published:** 2020-12-09

**Authors:** Xianjun Zhang, Stephen D. Carter, Jitin Singla, Kate L. White, Peter C. Butler, Raymond C. Stevens, Grant J. Jensen

**Affiliations:** 1 Department of Biological Sciences, Bridge Institute, USC Michelson Center for Convergent Bioscience, University of Southern California, Los Angeles, CA 90089, USA.; 2 Division of Biology and Biological Engineering, California Institute of Technology, Pasadena, CA 91125, USA.; 3 Larry Hillblom Islet Research Center, Department of Medicine, David Geffen School of Medicine, University of California, Los Angeles, Los Angeles, CA 90095, USA.; 4 Department of Chemistry, Bridge Institute, USC Michelson Center for Convergent Bioscience, University of Southern California, Los Angeles, CA 90089, USA.; 5 Howard Hughes Medical Institute (HHMI), California Institute of Technology, Pasadena, CA 91125, USA.

## Abstract

Subcellular neighborhoods, comprising specific ratios of organelles and proteins, serve a multitude of biological functions and are of particular importance in secretory cells. However, the role of subcellular neighborhoods in insulin vesicle maturation is poorly understood. Here, we present single-cell multiple distinct tomogram acquisitions of β cells for in situ visualization of distinct subcellular neighborhoods that are involved in the insulin vesicle secretory pathway. We propose that these neighborhoods play an essential role in the specific function of cellular material. In the regions where we observed insulin vesicles, a measurable increase in both the fraction of cellular volume occupied by vesicles and the average size (diameter) of the vesicles was apparent as sampling moved from the area near the nucleus toward the plasma membrane. These findings describe the important role of the nanometer-scale organization of subcellular neighborhoods on insulin vesicle maturation.

## Teaser

Multiple distinct tomograms of a single β cell enable investigation of the architecture of insulin vesicle neighborhoods.

## INTRODUCTION

Subcellular neighborhoods consist of groupings of specialized organelles and proteins that have one or more specific jobs to perform in the cell. The temporal and spatial distribution of these neighborhoods is essential for a multitude of biological functions, including protein production, maturation, and transport (*1*). Insulin is a peptide hormone produced by pancreatic β cells that is released when blood glucose levels rise (*2*). Insulin vesicle production and properly timed secretion are the most critical functions of β cells, with loss or misregulation leading to diabetes mellitus (*3*, *4*). Glucose-induced insulin secretion is a complex process that requires the endoplasmic reticulum (ER), Golgi, mitochondrial, cytoskeletal, and membrane fusion machinery work synergistically. However, fundamental questions remain about how these different components act together to orchestrate insulin secretion.

Three-dimensional (3D) electron tomography (ET), particularly of thin sections embedded in plastic, has been used to visualize the structures of β cell organelles (*5*–*8*). These studies have revealed, for instance, that mature insulin vesicles have a dark, electron-dense core surrounded by a halo (*9*, *10*). However, experimental artifacts from damage to or deformation of organelles introduced during sample preparation—a process that includes chemical fixation, dehydration, embedding, and staining (*5*, *7*, *11*)—have the potential to skew results and obscure important details. Thus, techniques to visualize the subcellular architecture in a near-native state are required to further our understanding of the context of the subcellular neighborhood interactions.

Cryo-ET has become standard for visualizing the 3D macromolecular organization of cells in an unperturbed context in situ by sample preservation through plunge freezing (*12*–*15*). Yet, because of the extreme radiation sensitivity of biological samples, montage cryo-ET has not been possible, and observations have been limited to just a miniscule fraction (hundredths of one percent) of the overall cell. We overcome these limitations by recording multiple tomograms from single cells, resulting in a census of subcellular structure at high resolution (*16*). By combining cryo-ET with cryo–focused ion beam (cryo-FIB) milling, we collected tens of tomograms from distinct regions along the secretory pathway of individual β cells to closely examine the subcellular neighborhoods and to visualize insulin vesicle maturation in situ at high resolution. Here, we provide perspective on the distinct roles of these subcellular constituents and their surrounding neighborhoods on insulin vesicle development and trafficking.

## RESULTS

### Mapping the β cell structure by single-cell multiple distinct tomogram acquisition

**Fig. 1:**
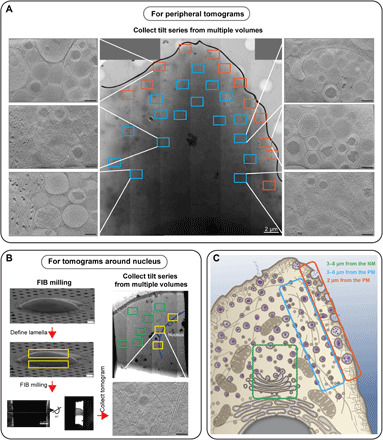
Single-cell multiple distinct tomogram acquisition with cryo-FIB milling and cryo-ET. (**A**) Multiple distinct tomograms acquired from the peripheral region of a single INS-1E cell with cryo-ET. 2D montage image of a cell grown on an electron microscopy (EM) grid. Boxes in blue (3 to 6 μm from the PM) and orange (2 μm from the PM) identify areas selected for tilt series image acquisition. Selected tomograms to illustrate the cellular ultrastructure from the indicated regions are shown (left and right). (**B**) Multiple distinct tomogram collection from a single lamella in the central region of the cell using cryo-FIB milling and cryo-ET. With the NM outlined in blue, boxes in green (3 to 8 μm from the NM) and yellow (at the NM) identify areas selected for tilt series image acquisition. (**C**) Cartoon map of the insulin vesicle secretory pathway and high-resolution cell structure. With this scheme, features including the nucleus, ER, Golgi, mitochondria, cytoskeleton, vesicles, and ribosomes revealed by tomograms can be mapped back into the cell (scale bars, 200 nm in tomographic slices).

We imaged INS-1E cells, a rat insulinoma pancreatic β cell line that is amenable to prolonged and consistent cell culturing and displays stable differentiated β cell phenotypes over a hundred cell passages. The glucose dose-response curve of INS-1E cells is similar to that of rat islets, suggesting that their insulin secretion pathways are comparable (*17*, *18*). To obtain a more complete picture of insulin secretion, we first collected a high-magnification 2D montage of the cell and used this as a map to target areas sufficient for cryo-ET imaging. We collected tens of tomograms from the thin peripheries of an individual cell, mapping the positions of the areas studied on a high-magnification view of the cell (Fig. 1A and fig. S1A). To image regions deeper in the interior of cells, we used cryo-FIB milling (*19*, *20*) to generate 180- to 250-nm-thick lamellae suitable for cryo-ET (Fig. 1B and fig. S1B). In 2D projection images from these lamellae, as a map, we could easily recognize the nucleus, ER, Golgi apparatus, and mitochondria. Multiple tomograms were recorded in different locations across the milled section of each lamella to develop a more complete view of the cell.

In total, 123 tomograms were recorded from the periphery of six cells, and 20 tomograms from a single β cell were selected for analysis. Thirty-seven tomograms of 10 lamellae from the interior of 10 cells were recorded, and 5 tomograms of separate lamellae from 3 distinct cells were selected for analysis (fig. S2 and table S1). Tomograms that show the stages of the phenotype we are interested in examining were selected and allowed us to collectively visualize the entire insulin vesicle secretion pathway. We consistently observed small vesicles around the ER and Golgi, presumably containing insulin and other proteins, as well as insulin-containing vesicles in the cytoplasm near the plasma membrane (PM) (Fig. 1C and movie S1).

### Characterization of the ER and Golgi apparatus

**Fig. 2:**
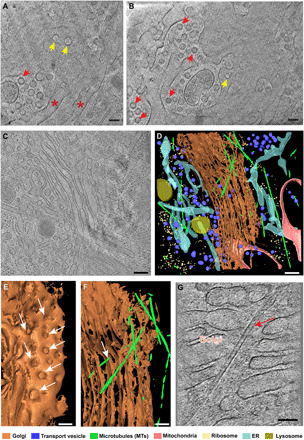
High-resolution structure of the ER and Golgi apparatus in INS-1E cells. (**A** and **B**) Slices through a tomogram showing the ER from different angles. Yellow arrows point to holes in the ER matrix, red asterisks show the tubular ER, and red arrows point to coat protein (COP)–coated vesicles. (**C**) Slice through a tomogram showing the Golgi apparatus. (**D**) 3D segmentation of the tomogram in (C) with color key shown at the bottom. (**E** and **F**) Close-up views of (D) showing 25- to 50-nm-diameter holes (white arrows) throughout the Golgi cisternae (E) and MTs passing through the Golgi body (F). (**G**) Representative tomographic slice showing an MT (red arrow) passing through the Golgi body [scale bars, 100 nm in (A), (B), and (E) to (G) and 200 nm in (C) and (D)].

The ER in Fig. 2A was observed 2 μm from the nuclear membrane (NM), and both the tubular ER matrices with three-way junctions and fenestrated ER sheets were evident, as previously observed with superresolution light-sheet microscopy (*21*). We observed coat protein (COP)–coated vesicles in different stages of maturation: partially coated budding or fusing profiles at the ER and fully coated COP vesicles in the cytoplasm at the periphery of the ER (Fig. 2B and fig. S3).

While the architecture of the Golgi apparatus has been well defined in previous studies (*6*, *22*, *23*), our results provide additional insights. The Golgi shown in the tomogram in Fig. 2C is representative of our observations and was recorded 4 μm from the NM. The overall architecture comprises multiple parallel flat cisternae and transport vesicles (Fig. 2, C and D, and movie S2). The number of cisternae is different in different species, and seven cisternae are apparent in Fig. 2C. While the *Chlamydomonas* Golgi has approximately nine cisternae (*23*), our mammalian INS-1E cells typically exhibited five to seven cisternae. The intracisternal protein array observed in the *Chlamydomonas* Golgi was not found in our mammalian INS-1E cells. The cisternae had 25- to 50-nm-diameter holes (Fig. 2E), which is consistent with previous findings (*24*). Unexpectedly, we found microtubules (MTs) passing through the Golgi apparatus in samples from several different cells (Fig. 2, F and G).

### Insulin vesicles and their subcellular neighborhoods

**Fig. 3:**
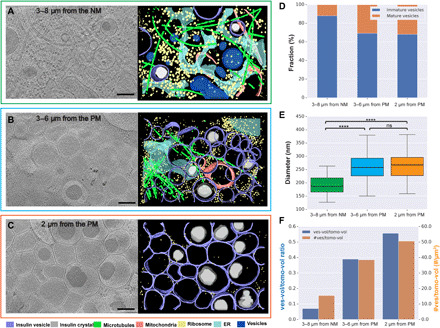
Visualizing insulin vesicle maturation and subcellular organization in situ. (**A**) Tomographic slice of an INS-1E cell thinned by cryo-FIB milling that is representative of region 1, the area between 3 and 8 μm from the NM (left). Segmentation of the tomographic data (right, color key below). (**B**) Representative tomographic slice of region 2, the area between 3 and 6 μm from the PM. (**C**) Representative tomographic slice of region 3, the area within 2 μm from the PM. (**D**) Relative percentage of mature and immature insulin vesicles quantified in the three regions of the cells studied. (**E**) Median diameter of insulin vesicles within each of the three regions in the cell; boxplot shows 25 to 75% quantile. ****P < 0.0001; ns, not significant in *t* test (and nonparametric tests). (**F**) Bar plot showing the fraction of insulin vesicle volume to tomogram volume (blue) and number of insulin vesicles per tomogram volume (orange) at the three regions [for (D) to (F): region 1 samples, *n* = 25 vesicles from five tomograms of five different cells; region 2 samples, *n* = 116 vesicles from 10 tomograms from one cell; region 3 samples, *n* = 130 vesicles from 10 tomograms from one cell] [scale bars, 200 nm in (A) to (C)].

Insulin vesicles are known to mature as they migrate toward the PM after release from the trans-Golgi. Light microscopy revealed that the cells were polarized and highly irregular in shape. The distance between the NM and PM in the horizontal plane ranged between 1 and 35 μm (fig. S4). We categorized this space into regions to evaluate differences in vesicle development across three subcellular microenvironments along the secretory pathway in INS-1E cells (*25*): region 1, just distal from the Golgi (approximately 3 to 8 μm from the NM examined in 5 tomograms of lamellae from three different cells); region 2, approximately midway between region 1 and the PM (3 to 6 μm from the PM in 10 tomograms of the periphery of one cell); and region 3, adjacent to the PM (within 2 μm of the PM in 10 tomograms of the periphery of one cell) (fig. S5). Both types of membrane landmarks, NM and PM, were needed as reference points because PMs were not usually visible in the atlases of lamellae, and NMs were not typically visible in atlases of the cell periphery. Representative tomograms from each of the three regions are shown in Fig. 3 (see also movies S3 to S5 and fig. S2). Distal to the Golgi, the ER formed ribbons and sheets (Fig. 3A) that were different from its more tubular morphology closer to the NM. Ribosomes were apparent in each of these different regions. However, the cellular content changed noticeably in areas closer to the cell’s periphery, the ultimate destination of the insulin vesicle for its final role in secretion. Our tomograms show that the cellular content in the region within 2 μm of the β cell’s PM is dominated by the presence of insulin vesicles, both immature and mature, as well as ribosomes.

Previous studies have shown that insulin crystallizes inside vesicles during the maturation process (*26*). We classified the vesicles in our tomograms as either mature or immature based on the presence or absence of a dense core. Both immature and mature insulin vesicles were seen in all three regions, but the percentage of mature insulin vesicles increased from 12% at the cell interior to 32% at the regions closer to the PM (Fig. 3D) in the cells that we studied. At the same time, the average diameter of the insulin vesicles increased from 196 ± 42 nm to 266 ± 52 nm (Fig. 3E). In the regions where insulin vesicles were observed in cells, they were packed with increasing density toward the periphery; the number of insulin vesicles per μm^3^ increased from 16 to 51 and the fraction of the volume occupied by insulin vesicles increased from 0.07 to 0.56 (Fig. 3F) across the three regions in our samples.

### Metal cluster deposit

**Fig. 4:**
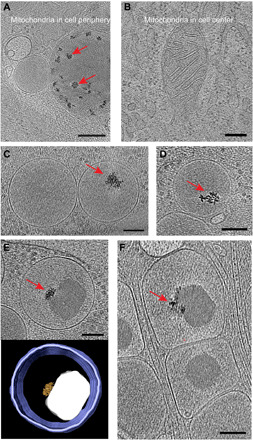
Metal clusters within mitochondria and insulin vesicles. (**A** and **B**) Tomographic slices showing mitochondria from the peripheral and central parts of the INS-1E cell, respectively. Red arrows indicate calcium stores. (**C** and **D**) Representative tomographic slices showing metal clusters (red arrows) in immature insulin vesicles. (**E** and **F**) Representative tomographic slices of metal clusters in mature insulin vesicles. Insulin vesicle membrane (purple), insulin crystal (gray), and metal cluster (gold) are segmented below [scale bars, 200 nm in (A) and (B) and 100 nm in (C) to (F)].

Solid-phase calcium stores are known to exist in the mitochondrial matrices of a variety of mammalian cell types (*27*). Here, we observed solid-phase calcium stores in the matrices of mitochondria in the periphery of β cells, but none in mitochondria in any of the 11 lamellae that we collected near the nucleus (Fig. 4, A and B). In ~10% of insulin vesicles, we observed one or two metal clusters, both within immature and mature vesicles (Fig. 4, C to F, and movie S6). These clusters contained 3- to 8-nm particles and ranged from 30 to 90 nm in diameter. Within immature insulin vesicles, the locations of these clusters appeared random, while within mature insulin vesicles they tended to be located very close or attached to the insulin crystal.

## DISCUSSION

Our single-cell multiple distinct tomogram study of β cells creates a biased census of components within the subcellular neighborhoods. To eliminate bias in future studies, regions/neighborhoods would be more appropriately sampled either randomly or systematically for more meaningful interpretation with better statistical representation and biological meaning. Furthermore, combining with another whole-cell imaging method, soft x-ray tomography, can give us much better and comprehensive knowledge about the cellular architecture of the whole-cell (*28*). These manually selected regions illustrate insulin vesicle maturation and trafficking in situ to serve as a pilot study toward deeper statistical analysis. We found that vesicles are larger, are packed more closely, and develop increasingly electron-dense crystalline cores as they move within the cell from the trans-Golgi to the PM. The average diameter of the insulin vesicles increased, revealing that vesicles were either fusing or more lipids were being added as they trafficked toward the PM. While it is not understood why β cells store so many insulin vesicles and release less than 5% of them quickly after glucose stimulation (*29*), our analysis suggests that this may be accounted for by the limited number of vesicles that are spatially located adjacent to the PM—there simply is insufficient space along the PM for more. The increasing density of insulin vesicles near the PM, as compared to their relative sparsity in more interior regions of the cell, appears to be part of a “healthy” cultured β cell’s preparation to have ample vesicles available to handle the multiple phases of insulin secretion over time following glucose stimulation. It has been shown that the first insulin vesicles to be synthesized are the first to be secreted (*30*), while older vesicles can be secreted under extreme stimulation or merged with lysosomes for recycling (*30*). While we observed differences in size, packing density (abundance), and insulin organization within the vesicles, it was not clear from our data whether these or other differences are the metrics that determine when vesicles are selected for either fusion or degradation. We speculate that the maturation and transport processes have evolved so that large, mature vesicles are abundant near the PM, where they can release large quantities of insulin.

Organization of subcellular neighborhoods within cells is key for stable cell architecture and function. For example, MTs play an important role in insulin vesicle trafficking as a path along which kinesin motors transport vesicles (*31*). We found multiple MTs penetrating the Golgi in our samples. The molecular mechanisms for how Golgi cisternae maintain separate yet parallel structure continues to be an area of study. There are many hypotheses about how Golgi membranes are tethered by Golgi reassembly stacking proteins (*32*). Our observation provides another explanation that MTs may play a role in keeping the Golgi cisternae close together, facilitating vesicle movement between cisternae, and/or localizing the Golgi within the cell.

The microenvironment or subcellular neighborhood appears to have an important role in the specific function of the organelle. Solid-phase calcium stores are known to provide a major ion reservoir in mitochondria for bioenergetics and signaling (*27*, *33*). However, the solid calcium that was observed in mitochondria near the periphery of cells, and was absent from mitochondria near the nucleus, suggests that mitochondria in different locations (neighborhoods) within the cell occupy different roles. Therefore, while mitochondria in the vicinity of the nucleus may be more responsible for energy generation, mitochondria at the cell’s periphery may be more involved in cell signaling and communication.

We observed metal clusters within insulin vesicles that were similar in appearance to the solid-phase calcium stores in the mitochondria. These metal clusters in insulin vesicles could be zinc repositories, because zinc is known to be essential for insulin crystallization in β cells (*26*, *34*, *35*). It was previously demonstrated in an in vivo assay that insulin does not crystallize when zinc transporters are knocked out (*26*). Further studies are required to definitively characterize which metals comprise these clusters, but they are likely released into the bloodstream along with insulin.

Using multiple tomograms from single cells allows the visualization of more of the subcellular architecture of an entire mammalian cell than has been possible previously (*36*, *37*). With the development of cryo-FIB lift-out techniques, we might soon be able to image lamellae from within actual tissue (*38*). Application of this method to studies of β cells within islets maintaining cell-cell contacts will provide context for how subcellular neighborhoods are altered in healthy and diseased states. These approaches can be used to study other cell types, such as α cells, neurons, and stem cells, and will open the door to understanding how differences in subcellular neighborhoods affect the specialized functions of various cell types throughout an organism.

## MATERIALS AND METHODS

### Experimental design

#### Cell culture

INS-1E cells [from P. Maechler’s laboratory at University of Geneva (*39*)] were cultured in T25 flasks in Addex Bio Optimized RPMI medium [RPMI modified to contain 2 mM l-glutamine, 10 mM Hepes, 1 mM sodium pyruvate, glucose (2000 mg/liter), and sodium bicarbonate (1500 mg/liter)] containing 10% fetal bovine serum and 0.05 mM β-mercaptoethanol. Cells were grown within 37°C incubators with 5% CO_2_ at 4 × 10^4^ cells/cm^2^ density and passed every 7 days, with one media change after 4 days.

#### Transmission electron microscopy grid preparation

Electron microscopy (EM) grids (200 mesh gold R2/2 London finder, Quantifoil Micro Tools, Germany) were coated with laminin (Sigma-Aldrich, L2020) for 30 min at room temperature. Cells were plated on grids at a density of 4 × 10^4^ cells/cm^2^. After 48-hour incubation, cells were treated with KREBS buffer [115 mM NaCl, 24 mM NaHCO_3_, 5 mM KCl, 1 mM MgCl_2_, 1 mM CaCl_2_, 10 mM Hepes, and 0.2% bovine serum albumin (BSA) (pH 7.4)] with no glucose and incubated for 30 min at 37°C. The buffer was exchanged to KREBS buffer supplemented with 25 mM glucose, and cells on grids were incubated for another 30 min at 37°C. EM grids were manually blotted and plunge-frozen in a liquid ethane/propane mixture using a Vitrobot Mark IV (FEI) (*40*). Grids were stored in liquid nitrogen until the experiment was conducted.

#### Cryo-FIB milling

EM grids were clipped into FEI AutoGrids and mounted into an FEI Versa 3D DualBeam FIB/SEM (scanning electron microscope) instrument with a cryo-transfer system (Quorum Technologies, PP3010T). Samples were coated with platinum in the Quorum preparation chamber (10 mA, 60 s) to improve conductivity (*20*). The vitrified samples were imaged at 5 to 10 keV with the SEM and milled with 30-keV gallium ions by scanning the regions of interest at a 20° tilt angle. A 0.3-nA beam current was used for rough milling, followed by lower currents during the thinning procedure (*41*). A 10-pA current was used for the polishing step to reach the final lamella thickness.

#### Cryo-ET and tomogram reconstruction

The frozen grids and lamellae were imaged in a 300-keV Titan Krios microscope (Thermo Fisher Scientific) equipped with an energy filter (Gatan) and a K3 Summit direct electron detector (Gatan). We first collected a high-magnification 2D montage of the cell periphery using SerialEM (*42*) and used this as a map to identify areas sufficient for cryo-ET imaging. We selected regions manually from our high-magnification montage that lacked ice contamination and were sufficiently electron-transparent for detailed analysis. We also avoided collecting on neighboring areas of the cell, which had already been exposed to electrons by previous steps. Tilt series were acquired with 2° steps between −60° and +60° (in two halves, separated at 0°). Individual tilt series were recorded at a magnification of ×26,000 or ×33,000 and a defocus of −6 to −10 μm. The cumulative dose of each tilt series was between 80 and 150 e^−^/Å^2^. Tilt series were aligned and binned by 4 using the IMOD software package (*43*), and 3D reconstructions were calculated using the simultaneous iterative reconstruction technique implemented in the IMOD software package (*44*) or weighted back-projection using IMOD.

#### Tomogram visualization and segmentation

Segmentation was performed with Amira software (Thermo Fisher Scientific). Insulin vesicles, insulin crystals, mitochondria, and ER were segmented manually using the thresholding tool. Golgi membranes were automatically segmented using the TomoSegMemTV package (*45*) and refined manually using Amira (Thermo Fisher Scientific). MTs and ribosomes were segmented using EMAN2 (*46*).

#### Fluorescent light microscopy

Cells were rinsed with Dulbecco’s phosphate-buffered saline (DPBS) (Thermo Fisher Scientific, 14190144) and incubated for 30 min at 4°C with the chemical Golgi dye, 5 μM ceramide–BSA complexes (Thermo Fisher Scientific, B-22650). The samples were then rinsed several times with DPBS and incubated in a fresh medium at 37°C for a further 30 min. The sample was imaged using a 60× extra-long-working-distance air objective [Nikon CFI S Plan Fluor ELWD 60× NA (numerical aperture) 0.7 WD (working distance) 2.62 to 1.8 mm]. Images were recorded using the NIS-Elements software from AutoQuant (Nikon Instruments Inc., Melville, NY).

### Statistical analysis

#### Statistics of diameter and volume of insulin vesicles

Twenty peripheral tomograms containing notable insulin vesicles were selected from a representative cell that had plenty of insulin vesicles for analysis. Five tomograms with notable insulin vesicles from three lamellae were selected for analysis. Computing the diameter and volume of the vesicles was difficult by direct segmentation because the complete membrane of vesicles was not visible in tomograms due to the missing wedge. As most of the vesicles appeared to be spherical, we estimated the diameter and volume from a central cross section of the vesicle as

$$\begin{array}{c} d=2*\sqrt{\frac{A}{\pi }}\\ V=\frac{\pi d^{3}}{6} \end{array}$$

where *d* and *V* are the diameter and volume of the insulin vesicle, respectively, and *A* is the area of the vesicle cross section.

Because we observed multiple vesicles at the three spatial locations described in Results (i.e., region 1, 3 to 8 μm from NM; region 2, 3 to 6 μm from PM; and region 3, 2 μm from PM), we report the average diameter as well as the complete variance in the box plot in Fig. 3E.

## Supplementary Material

http://advances.sciencemag.org/cgi/content/full/6/50/eabc8258/DC1

Movie S1

Movie S2

Movie S3

Movie S4

Movie S5

Movie S6

Adobe PDF - abc8258_SM.pdf

Visualizing insulin vesicle neighborhoods in β cells by cryo–electron tomography

## SUPPLEMENTARY MATERIALS

Supplementary material for this article is available at http://advances.sciencemag.org/cgi/content/full/6/50/eabc8258/DC1

## Appendix

View/request a protocol for this paper from *Bio-protocol*.
