# Supplementary material for: Visualizing insulin vesicle neighborhoods in β cells by cryo–electron tomography
Source: Sci Adv. 2020 Dec 9;6(50):eabc8258. doi: 10.1126/sciadv.abc8258 (PMC7725471; doi:10.1126/sciadv.abc8258)
Supplement: http://advances.sciencemag.org/cgi/content/full/6/50/eabc8258/DC1 [file supp_6_50_eabc8258__index.html]

Science Advances | Science AdvancesAAASSearchScience AdvancesMenu

## Supplementary Materials

# Visualizing insulin vesicle neighborhoods in β cells by cryo–electron tomography

Xianjun Zhang, Stephen D. Carter, Jitin Singla, Kate L. White, Peter C. Butler, Raymond C. Stevens, Grant J. Jensen

Download Supplement

**The PDF file includes:**

- Figs. S1 to S5
- Table S1
- Legends for movies S1 to S6

**Other Supplementary Material for this manuscript includes the following:**

- Movie S1
- Movie S2
- Movie S3
- Movie S4
- Movie S5
- Movie S6

**Files in this Data Supplement:**

- Adobe PDF - abc8258\_SM.pdf
- abc8258\_Movie\_S1.mp4
- abc8258\_Movie\_S2.mp4
- abc8258\_Movie\_S3.mp4
- abc8258\_Movie\_S4.mp4
- abc8258\_Movie\_S5.mp4
- abc8258\_Movie\_S6.mp4
