# Supplementary material for: Visualizing insulin vesicle neighborhoods in β cells by cryo–electron tomography
Source: Sci Adv. 2020 Dec 9;6(50):eabc8258. doi: 10.1126/sciadv.abc8258 (PMC7725471; doi:10.1126/sciadv.abc8258)
Supplement: http://advances.sciencemag.org/cgi/content/full/6/50/eabc8258/DC1 [file supp_6_50_eabc8258__1.pdf]

Science Advances
AAAS

advances.sciencemag.org/cgi/content/full/6/50/eabc8258/DC1

Supplementary Materials for

Visualizing insulin vesicle neighborhoods in β cells by cryo–electron tomography

Xianjun Zhang, Stephen D. Carter, Jitin Singla, Kate L. White, Peter C. Butler, Raymond C. Stevens*, Grant J. Jensen*

*Corresponding author. Email: stevens@usc.edu (R.C.S.); jensen@caltech.edu (G.J.J.)

Published 9 December 2020, *Sci. Adv.* **6**, eabc8258 (2020)
DOI: 10.1126/sciadv.abc8258

The PDF file includes:

Figs. S1 to S5
Table S1
Legends for movies S1 to S6

Other Supplementary Material for this manuscript includes the following:

(available at advances.sciencemag.org/cgi/content/full/6/50/eabc8258/DC1)

Movies S1 to S6

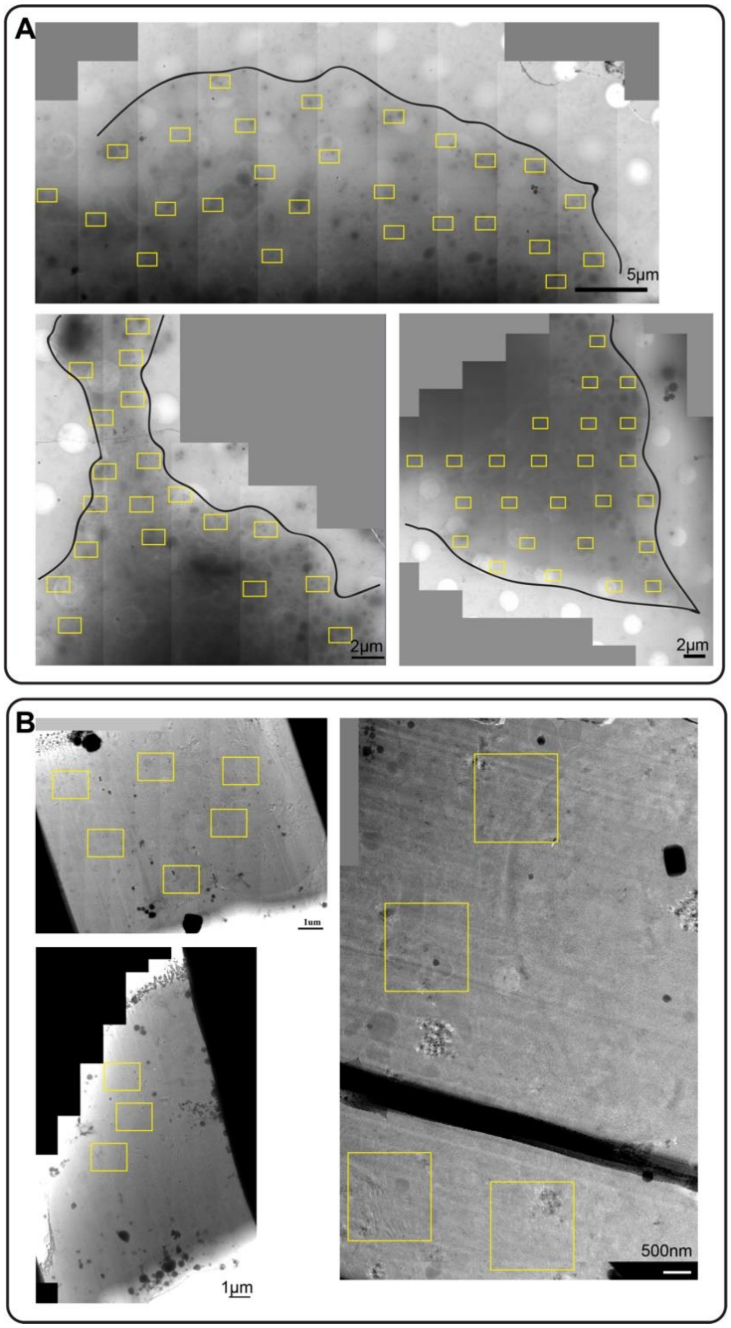

Fig. S1. Further examples of cells periphery and lamellae.

(A) Additional examples of representative cells showing areas targeted for data collection. **(B)** 2D projection images of lamellae. Locations of tomograms taken from cells and lamellae are shown with yellow boxes.

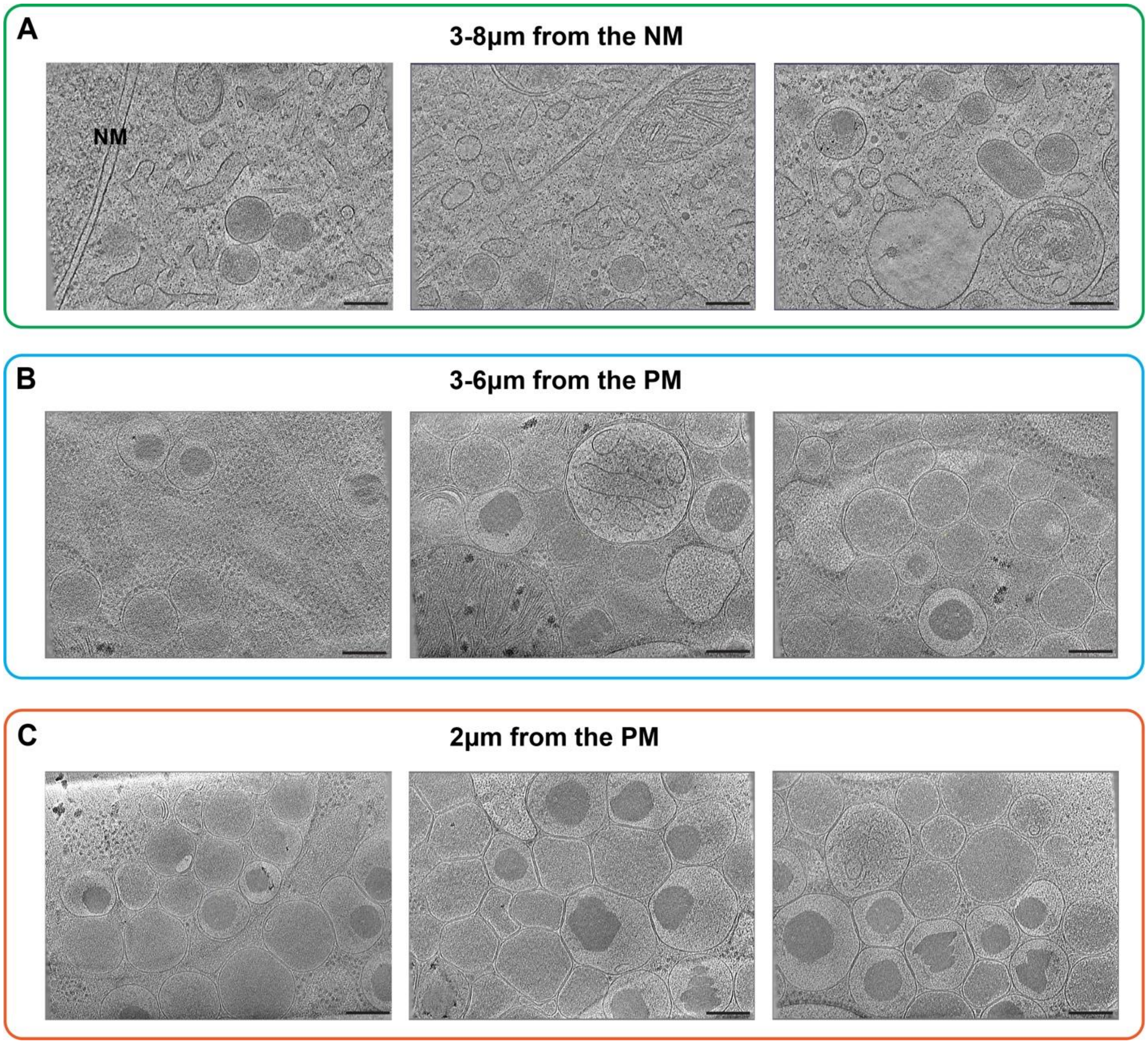

Fig. S2. More examples of tomographic slices from each region.

(A) Representative tomographic slices of Region 1, the area between 3-8 µm from the NM. **(B)** Representative tomographic slices of Region 2, the area between 3-6 µm from the PM. **(C)** Representative tomographic slices of Region 3, the area within 2 µm of the PM. (Scale bars: 200 nm)

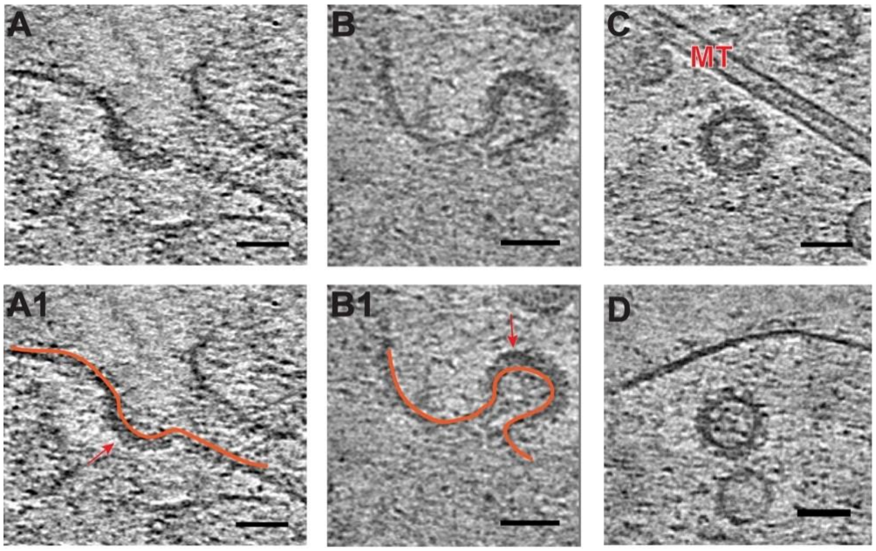

Fig. S3. COP-coated vesicles in different stages of maturation.

(A, B) Tomographic slices of COP-coated buds in different stages of maturation. **A1, B1,** Segmentation of membrane (orange line). Red arrows point to coat proteins. **(C, D)** Cytoplasmic fully COP-coated vesicles present at the periphery of the ER. MT: microtubules. (Scale bars: 50 nm)

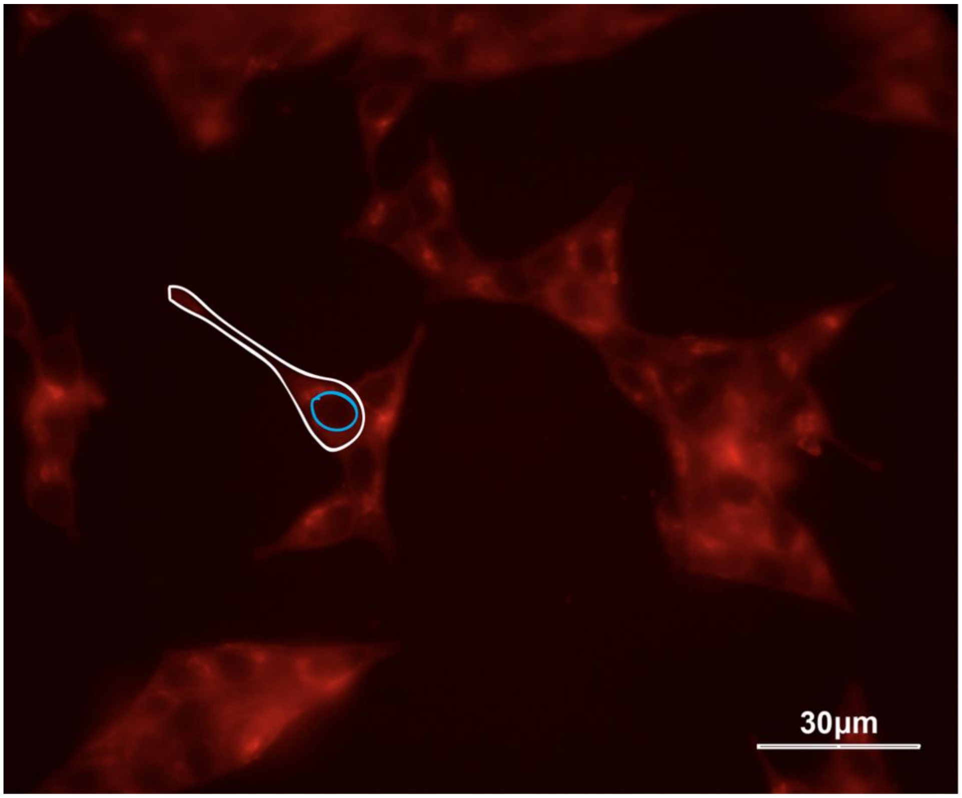

Fig. S4. Fluorescent light microscope image of INS-1E cells

Fluorescent light microscope image of INS-1E cells stained with chemical Golgi dye. The PM and NM are showed with white and blue rings, respectively.

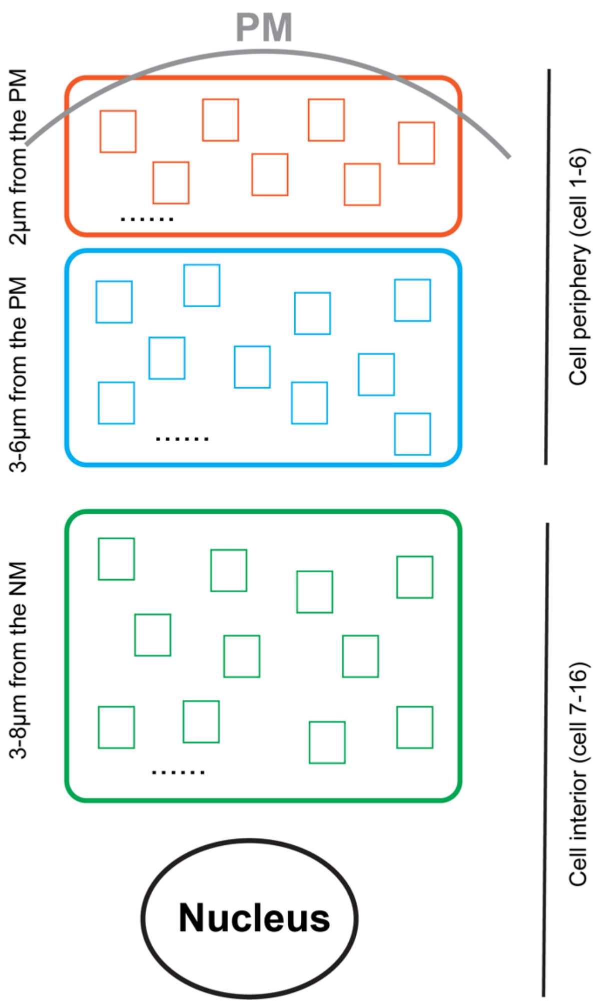

Fig. S5. Schematic of data collection in different regions within the cell.

Region 1 (green) - just past the Golgi (between 3 to 8 μm from the NM in tomograms of lamellae). Region 2 (blue) - approximately mid-way between region 1 and PM (3 to 6 μm from the PM in tomograms of the cell periphery); and Region 3, orange, adjacent to the PM (within 2 μm of the PM in tomograms of the cell periphery). Tomograms taken in each region are indicated by small boxes.

Table S1. Multiple distinct tomograms acquisition from individual cells.

Cell ID	Locations	Number of tomograms collected
1	Cell periphery	11
2	Cell periphery	15
3	Cell periphery	36
4	Cell periphery	18
5	Cell periphery	26
6	Cell periphery	17
7	Cell interior (lamella 1)	2
8	Cell interior (lamella 2)	3
9	Cell interior (lamella 3)	3
10	Cell interior (lamella 4)	3
11	Cell interior (lamella 5)	2
12	Cell interior (lamella 6)	9
13	Cell interior (lamella 7)	2
14	Cell interior (lamella 8)	4
15	Cell interior (lamella 9)	7
16	Cell interior (lamella 10)	2

Movie S1 to S6

Movie S1. Multiple distinct tomograms acquisition from an individual cell.

2D montage image of an INS-1E cell grown on an EM grid with yellow boxes showing areas selected for tilt series acquisition. Black line indicates location of PM. Tomograms at various locations are highlighted in turn, scrolling through the tomographic volume in Z.

Movie S2. Cryo-ET of native Golgi.

The video slices up and down in Z through the tomographic volume, then reveals the 3D segmentation with structures identified as: Golgi: gold; transport vesicles: purple; MTs: green; mitochondria: pink; ribosomes: yellow; and ER: cyan.

Movie S3. Representative tomogram from the area between 3 to 8 µm of the NM.

The video slices up and down in Z through the tomographic volume, then reveals the 3D segmentation with structures identified as: insulin vesicle membrane: purple; insulin crystals: grey; MTs: green; mitochondria: pink; ribosomes: yellow; ER: cyan; and unknown vesicles: dark blue.

Movie S4. Representative tomogram from the area between 3 to 6 µm of the PM.

The video slices up and down in Z through the tomographic volume, then reveals the 3D segmentation with structures identified as: insulin vesicle membrane: purple; insulin crystals: grey; MTs: green; mitochondria: pink; ribosomes: yellow; and ER: cyan.

Movie S5. Representative tomogram from the area at 2 µm from the PM.

The video slices up and down in Z through the tomographic volume, then reveals the 3D segmentation with structures identified as: insulin vesicle membrane: purple; insulin crystals: grey; and ribosomes: yellow.

Movie S6. Metal cluster deposit in insulin vesicle.

The video slices up and down in Z through the tomographic volume, then reveals the 3D segmentation of the metal cluster deposit with structures identified as: insulin vesicle membrane: purple; insulin crystal: grey; and metal cluster: gold.
